# Anabolic–androgenic steroid use is associated with psychopathy, risk-taking, anger, and physical problems

**DOI:** 10.1038/s41598-022-13048-w

**Published:** 2022-06-01

**Authors:** Bryan S. Nelson, Tom Hildebrandt, Pascal Wallisch

**Affiliations:** 1 Department of Psychology, New York University, New York, NY USA; 2 Department of Psychiatry, Icahn School of Medicine at Mount Sinai, New York, NY USA

**Keywords:** Psychology, Human behaviour

## Abstract

Previous research has uncovered medical and psychological effects of anabolic–androgenic steroid (AAS) use, but the specific relationship between AAS use and risk-taking behaviors as well as between AAS use and psychopathic tendencies remains understudied. To explore these potential relationships, we anonymously recruited 492 biologically male, self-identified bodybuilders (median age 22; range 18–47 years) from online bodybuilding fora to complete an online survey on Appearance and Performance Enhancing Drug (APED) use, psychological traits, lifestyle choices, and health behaviors. We computed odds ratios and 95% confidence intervals using logistic regression, adjusting for age, race, education, exercise frequency, caloric intake, and lean BMI. Bodybuilders with a prior history of AAS use exhibited heightened odds of psychopathic traits, sexual and substance use risk-taking behaviors, anger problems, and physical problems compared to those with no prior history of AAS use. This study is among the first to directly assess psychopathy within AAS users. Our results on risk-taking, anger problems, and physical problems are consistent with prior AAS research as well as with existing frameworks of AAS use as a risk behavior. Future research should focus on ascertaining causality, specifically whether psychopathy is a risk associated with or a result of AAS use.

## Introduction

An estimated 6% of males globally^1^ (including 2.9–4 million Americans^2^) have used anabolic–androgenic steroids (AAS) such as methyltestosterone, danazol, and oxandrolone, which are a series of synthetic variants of the male sex hormone testosterone that increase lean muscle protein synthesis without increasing fat mass^3,4^. Although there are medical uses such as for AIDS-related wasting syndrome^5^, AAS are commonly used by individuals for the purposes of bodybuilding and appearance modification^2,3,6^. In these cases, doses are commonly 10 to 100 times higher than clinical doses and are typically “cycled” intermittently (i.e., used for a few months, stopped to minimize the stress that AAS impart on the body, then resumed shortly thereafter)^3,7^. AAS have a 30% dependence rate among long-term users, higher than many other prescription or illicit drugs such as cocaine and have been linked to medical issues such as liver and kidney damage, cardiovascular problems, testicular atrophy, infertility, hair loss, and gynecomastia^2,3,7–10^. AAS use is strongly associated with other substance abuse^8,9,11,12^, and users often exhibit negative, although idiosyncratic, psychological issues^8,13–17^. Some users report delusions of grandeur and invincibility, while others experience depression and various mood disturbances^8,18–20^. As dosage increases, AAS users may become impulsive, moody, aggressive, or even violent^9,18,19,21–27^. Recent neurobiological studies have focused on effects of AAS on central nervous system functions such as cognition, anxiety, depression, and aggression^10,28,29^. In recent imaging studies, AAS use was associated with cortex thinning as well as decreased gray matter and increased right amygdala volume^30–32^. AAS use seems to accelerate brain aging through oxidative stress and apoptosis^33–35^, is associated with lower cognitive function^36,37^, and may disrupt normal neuronal function in the forebrain, which can increase anxiety and aggressiveness and diminish inhibitory control^10,38–45^. Increased depression has been frequently observed during AAS withdrawal^32,46^.

One area that remains understudied among AAS users is psychopathy, a personality disorder characterized by shallow emotional affect, lack of empathy, and antisocial behavior^47–49^. Psychopathy research has frequently associated psychopathy with violence, repeated imprisonment, disrespect for authority, and substance misuse/abuse^48–55^. There is growing evidence that AAS use may be associated with psychopathy, including a direct association between AAS and psychopathy in an Iranian sample^56^ as well as numerous reports of associations between AAS use and violent crime or “roid rage”^19,21–23,25,27,57^. Prior studies examining AAS use and elements of the “Dark Triad” and “Big Five” personality traits suggest that the relationship between AAS use and both violence and risk-behaviors may be due to self-regulatory deficits and low conscientiousness, and that AAS use is predicted by narcissism, low agreeableness, neuroticism, impulsivity, and inability to delay gratification^56,58^. Hauger et al.^28^ recently identified significantly lower emotion recognition in AAS dependent users compared to AAS non-using weightlifters, suggesting that this lower emotion recognition may contribute to the higher frequencies of antisocial traits that AAS users have previously reported^59,60^. Antisocial personality disorder, which is characterized by the disregard for laws and norms, irritability, and the failure to regard the safety of self and others^61^ has been suggested as the mechanism that underlies the link between AAS use and aggression^3,9,60,62,63^. Conceptually, there are overlaps between antisocial personality disorder and psychopathy^64^. We therefore argue that psychopathic traits among AAS users are worth exploring.

Thus, the present study assessed whether AAS users were more likely than nonusers to exhibit psychopathic traits, risk-taking behaviors such as sharing needles, anger problems such as getting into altercations, emotional problems such as panic attacks and depression, cognitive problems such as difficulty remembering, and physical problems such as hair loss. We hypothesized that AAS users would display heightened odds of psychopathic traits, substance use risk-taking behaviors, sexual risk-taking behaviors, anger problems, emotional stability problems, cognitive problems, depressive symptoms, anxiety symptoms, impulsivity symptoms, and physical problems, although we recognize that many of these traits are highly idiosyncratic in nature. Finally, we hypothesized there is a dose-dependent relationship between these traits and the variety of substances used as well as the number of cycles.

## Method

### Participants and procedure

This study was approved by the NYU Committee on Activities Involving Human Subjects and we conducted in accordance with the Declaration of Helsinki principles. We anonymously recruited a large online sample of 492 (Mean age = 22.9, SD age = 4.3) adult biologically male bodybuilders and asked them questions about their Appearance and Performance Enhancing Drug (APED) use (if any), exercise and dietary habits, psychological states, risk-taking behaviors, and any physical problems they might have experienced. The anonymous internet survey was posted to online fitness fora in fall 2015. All participants provided informed consent prior to their participation. Participants had the option to enter an online raffle for one of twenty $50 Amazon gift cards, which were distributed via email.

### Measures

The following subsections are presented in the same order as the online survey.

#### Diet and exercise

Participants reported how often they had exercised in the past month (every day, most days, some days, very rarely/never) and rated their caloric intake in the past month on a 5-point ordinal scale (1 = extreme restriction of calories, 5 = extreme over-consumption of calories). We measured caloric intake in terms of restriction, maintenance, or surplus rather than total calories per day because participants likely vary in caloric requirements (i.e., 3000 cal/day may be a surplus for some but a deficit for others).

#### Appearance and performance enhancing drugs

Each participant indicated whether he had ever used oral, injectable, or topical AAS (“yes, currently,” “yes, formerly,” “no, but considered taking,” “no, never considered taking” for each). Additionally, participants reported how many AAS cycles they had completed and responded whether they had ever used the following APEDs (each with “yes”/”no” options): Testosterone, Dianabol (Methandrostenolone), Deca Durabolin (Nandrolone Decanoate), Winstrol (Stanozolol), Anadrol (Oxymetholone), Human Growth Hormone (Somatropin), Synthol, Anti-Estrogens, Fat Burners (Insulin, Clenbuterol, Cytomel, Cynomel), Trenbolone, or Anavar.

#### Self-reported events

Participants rated each of the following items as “yes, currently,” “yes, formerly,” or “no, never”.

***General events*** Participants self-reported whether they experienced the following events: depression, increased number of mood swings, getting into altercations, panic attacks, irritability, lack of frustration tolerance, aggression, difficulty focusing, racing thoughts, difficulty making decisions, difficulty remembering, suicidal thoughts, acne, trouble sleeping, water retention, hair loss, changes in appetite, and heart problems.

***Risk-taking behavior*** Participants indicated whether they had engaged in or experienced the following: unprotected sex, sex with multiple partners, sexually transmitted disease or infection (STD), sharing needles, reusing needles, using stimulants without prescription (such as crack, powdered cocaine, methamphetamine, amphetamine, or ecstasy [MDMA]), using opiates without prescription (such as heroin, morphine, codeine, or Oxycontin), using hallucinogens without prescription (such as LSD, mescaline, and psilocybin), using depressants without prescription (such as Valium, Xanax, Librium, and barbiturates), drinking alcohol, smoking tobacco, and smoking marijuana.

#### Impulsivity

We used the Barratt Impulsiveness Scale to quantify impulsivity (BIS-11)^65^. Participants responded to 30 statements such as “I often have extraneous thoughts” using a 4-point ordinal rating scale (1 = rarely/never, 4 = almost always/always). The BIS-11 displayed strong reliability in this sample (Cronbach’s α = 0.84).

#### Psychopathic traits

We employed the Levenson Self-Report Psychopathy Scale (LSRP) to assess psychopathy^66^. The scale has 26 items graded on a 5-point Likert scale (1 = strongly disagree, 5 = strongly agree) and was strongly reliable in this sample (Cronbach’s α = 0.88).

#### Anxiety

We assessed anxiety with the Generalized Anxiety Disorder 7-item Scale (GAD-7)^67^. Participants responded to each of the seven items such as “being so restless it is hard to sit still” on a 4-point ordinal rating scale (0 = not at all, 3 = nearly every day). The GAD-7 displayed excellent internal consistency (Cronbach’s α = 0.89). Possible scores range from 0 to 21.

#### Depression

We included the 10-item Center for Epidemiologic Studies Short Depression Scale (CES-D 10)^68^ to measure depression. Participants rated statements such as “I felt lonely” on a 4-point ordinal rating scale (0 = rarely or none of the time, 3 = all the time). The CES-D 10 was highly reliable (Cronbach’s α = 0.82), with possible scores ranging from 0 to 30.

#### Aggravation

Participants responded to the 7-item aggravation subscale of the State Hostility Scale^69,70^. In the subscale, participants rate possible descriptions of their current mood (e.g., “stormy” or “vexed”) on a 5-point Likert scale (1 = strongly disagree, 5 = strongly agree). The aggravation subscale of the State Hostility Scale had strong reliability (Cronbach’s α = 0.90).

#### Demographic questions

Lastly, participants reported their age (years), height (inches), weight (pounds), body fat percentage, racial background, and level of education.

### Statistical analysis

The survey was convenience sampled, with no pre-specified sample size or power calculation. For our primary analysis, we grouped participants who responded “yes, currently” or “yes, formerly” to having used AAS (oral, injectable, or topical) as AAS users (n = 154, 31.3%). We considered those who responded “no, but considered taking” or “no, never considered taking” to be AAS nonusers (n = 338, 68.7%). We also conducted a secondary analysis using all four categories (current AAS users (n = 121, 24.6%); former AAS users (n = 33, 6.7%); AAS nonuser, considered using (n = 200, 40.7%); AAS nonuser, never considered using (n = 138, 28.0%)).

Both AAS cycle experience and APED variety were self-reported. APED variety was the number of different APED types used (the number each participant responded “yes” to taking of Testosterone, Dianabol (Methandrostenolone), Deca Durabolin (Nandrolone Decanoate), Winstrol (Stanozolol), Anadrol (Oxymetholone), Human Growth Hormone (Somatropin), Synthol, Anti-Estrogens, Fat Burners (Insulin, Clenbuterol, Cytomel, Cynomel), Trenbolone, and Anavar). AAS cycle experience was the number of AAS cycles participants reported. If the participant was an AAS nonuser, then both APED variety and AAS cycle experience were scored as 0.

We grouped traits of interest into the following categories: psychopathic traits, substance use risk-taking behavior, sexual risk-taking behavior, anger problems, emotional stability problems, cognitive problems, depressive symptoms, anxiety symptoms, impulsivity symptoms, and physical problems. Following Brinkley et al.^71^, we considered participants in the top third of the LSRP distribution to have psychopathic traits. We considered any participant that reported sharing needles, reusing needles, hallucinogen use, stimulant use, depressant use, or opiate use as engaging in substance use risk-taking. Similarly, any participant that reported an STD, engaging in unprotected sex, or having multiple sexual partners was categorized as having sexual risk-taking behavior. Any participant scoring in the top half of the aggravation subscale of the State Hostility Scale, reporting physical altercations, or reporting increased aggression was categorized as having anger problems. Participants who reported mood swings, lower frustration tolerance, or irritability were considered to have emotional stability problems while participants with difficulty remembering, difficulty focusing, or trouble making decisions were considered to have cognitive problems. We considered participants with depressive symptoms as those that reported suicidal thoughts, reported increased depression, or had a CES-D 10 score greater than 10 (the established cut point^68^). Those with anxiety symptoms either had a GAD-7 score greater than the established cut point^67^ of 8 or reported panic attacks. A participant who reported racing thoughts or who scored in the top half of the Barratt Impulsiveness Scale was considered to have impulsivity symptoms. Finally, we considered participants to have physical problems if they reported heart problems, appetite changes, water retention, acne, or hair loss.

We used logistic regression to assess possible associations between these traits of interest and AAS use, number of AAS cycles, and variety of APEDs used. We computed odds ratios (OR) with 95% confidence intervals (CI). All analyses adjusted for age, race, education, exercise frequency, caloric intake, and lean BMI. Age, race, and education were included as basic demographic variables, while exercise frequency, caloric intake, and lean BMI were included to account for differences in bodybuilding goals, success, and dedication. We chose to calculate lean BMI to assess how muscular participants were. We used the standard (kg/m^2^) BMI formula but used each participant’s lean bodyweight instead of his total bodyweight. Lean body weight was calculated by using each participant’s self-reported body fat percentage to determine how much he weighed excluding his body fat (weight in kg * (100%-bodyfat%)). Given that both psychopathy and AAS use are associated with illicit drug use^21^, we conducted a post hoc subgroup analysis among participants without history of polysubstance use (3 or more different drug classes) to ensure any association between AAS use and psychopathic traits was not confounded by polysubstance use. All analyses were conducted in R (version 3.5.1).

### Ethics approval

This study was performed in line with the principles of the Declaration of Helsinki. Approval was granted by the Ethics Committee of New York University.

### Consent to participate

Participants provided informed consent prior to their participation in this anonymous internet survey.

## Results

Participant characteristics are listed in Table 1. Most participants were younger than 25 years old (56.5% of AAS users; 79.0% of AAS nonusers), white (85.7% of AAS users; 77.5% of AAS nonusers), and had education beyond high school (75.3% of AAS users; 59.1% of AAS nonusers). The majority in each group exercised most days of the week (79.2% of AAS users; 74.8% of AAS nonusers) and were attempting to gain weight (51.3% of AAS users; 51.2% of AAS nonusers). For AAS users and nonusers, the median (Q1-Q3) lean BMI was 23.6 (22.3–25.4) and 21.6 (20.3–23.3) kg/m^2^. AAS users began use at a median (Q1-Q3) of 21 (20–24) years, had completed 2 (1–3) AAS cycles, and used 4 (2–5) different APED types; 78.6% (121/154) were current AAS users. Among AAS nonusers, 59.2% (200/338) had considered using AAS.

**Table 1:** Participant characteristics.

	Current AAS User (n = 121)	Former AAS User (n = 33)	AAS User (n = 154)	AAS Nonuser, Considered Using (n = 200)	AAS Nonuser, Never Considered Using (n = 138)	AAS Nonuser (n = 338)
**N (%)**						
**Age**						
Younger than 25	74 (61.2)	13 (39.4)	**87 (56.5)**	162 (81.0)	105 (76.1)	**267 (79.0)**
25 or Older	47 (38.8)	20 (60.6)	**67 (43.5)**	38 (19.0)	33 (23.9)	**71 (21.0)**
**Race**						
White	102 (84.3)	30 (90.9)	**132 (85.7)**	165 (82.5)	97 (70.3)	**262 (77.5)**
Non-White	19 (15.7)	3 (9.1)	**22 (14.3)**	35 (17.5)	41 (29.7)	**76 (22.5)**
**Level of Education**						
High School or Less	32 (26.4)	6 (18.2)	**38 (24.7)**	79 (39.7)	59 (42.8)	**138 (40.9)**
Greater than High School	89 (73.6)	27 (81.8)	**116 (75.3)**	120 (60.3)	79 (57.2)	**199 (59.1)**
**Exercise Frequency**						
Every Day	25 (20.7)	1 (3.0)	**26 (16.9)**	32 (16.1)	20 (14.5)	**52 (15.4)**
Most Days	92 (76.0)	30 (90.9)	**122 (79.2)**	154 (77.4)	98 (71.0)	**252 (74.8)**
Some Days	4 (3.3)	2 (6.1)	**6 (3.9)**	13 (6.5)	20 (14.5)	**33 (9.8)**
**Caloric Intake**						
Weight Gain	68 (56.2)	11 (33.3)	**79 (51.3)**	111 (55.5)	62 (44.9)	**173 (51.2)**
Maintenance	14 (11.6)	11 (33.3)	**25 (16.2)**	45 (22.5)	36 (26.1)	**81 (24.0)**
Weight Loss	39 (32.2)	11 (33.3)	**50 (32.5)**	44 (22.0)	40 (29.0)	**84 (24.9)**
**Median (Q1-Q3)**						
Lean BMI	23.5 (22.2–25.4)	23.8 (23.2–25.4)	**23.6 (22.3–25.4)**	21.7 (20.5–23.5)	21.5 (19.8–22.9)	**21.6 (20.3–23.3)**
Age Began AAS	21 (20–24)	21 (19–23.5)	**21 (20–24)**	–	–	–
Number of Different APED Types Used	4 (2–6)	3 (1–4)	**4 (2–5)**	0	0	**0**
Number of AAS Cycles	2 (1–4)	1 (1–2)	**2 (1–3)**	0	0	**0**

Overall columns for primary analysis (AAS User, AAS Nonuser) are in bold.

Tables 2 and 3 summarize traits of interest and specific substance use risk-taking behaviors by AAS use status; 25.8% (39/154) of AAS users and 10.2% (34/338) of AAS nonusers had a history of polysubstance use. AAS users had over twice the odds of exhibiting psychopathic traits (OR = 2.50, 95% CI 1.52–4.15), over three times the odds of engaging in substance use risk-taking behaviors (OR = 3.10, 95% CI 1.97–4.93), nearly twice the odds of engaging in sexual risk-taking behaviors (OR = 1.79, 95% CI 1.01–3.26), nearly twice the odds of experiencing anger problems (OR = 1.71, 95% CI 1.02–2.95), and over twice the odds of exhibiting physical problems (OR = 2.23, 95% CI 1.16–4.51) compared to AAS nonusers (Table 4). In a post hoc subgroup analysis, AAS users without history of polysubstance use had higher odds of psychopathic traits compared to nonusers without history of polysubstance use (OR = 2.73, 95% CI 1.54–4.90).

**Table 2:** Traits of interest by AAS use status.

	Current AAS User (n = 121)	Former AAS User (n = 33)	AAS User (n = 154)	AAS Nonuser, Considered Using (n = 200)	AAS Nonuser, Never Considered Using (n = 138)	AAS Nonuser (n = 338)
**N (%)**						
**Psychopathic Traits**						
Yes	49 (41.9)	7 (22.6)	**56 (37.8)**	68 (34.9)	28 (21.7)	**96 (29.6)**
No	68 (58.1)	24 (77.4)	**92 (62.2)**	127 (65.1)	101 (78.3)	**228 (70.4)**
**Substance Use Risk-Taking Behavior**						
Yes	78 (65.0)	17 (51.5)	**95 (62.1)**	88 (44.9)	28 (20.9)	**116 (35.2)**
No	42 (35.0)	16 (48.5)	**58 (37.9)**	108 (55.1)	106 (79.1)	**214 (64.8)**
**Sexual Risk-Taking Behavior**						
Yes	105 (86.8)	29 (87.9)	**134 (87.0)**	157 (78.5)	74 (54.0)	**231 (68.5)**
No	16 (13.2)	4 (12.1)	**20 (13.0)**	43 (21.5)	63 (46.0)	**106 (31.5)**
Anger Problems						
Yes	100 (82.6)	24 (75.0)	**124 (81.0)**	161 (81.3)	83 (61.9)	**244 (73.5)**
No	21 (17.4)	8 (25.0)	**29 (19.0)**	37 (18.7)	51 (38.1)	**88 (26.5)**
**Emotional Stability Problems**						
Yes	86 (71.1)	17 (53.1)	**103 (67.3)**	131 (65.5)	71 (53.8)	**202 (60.8)**
No	35 (28.9)	15 (46.9)	**50 (32.7)**	69 (34.5)	61 (46.2)	**130 (39.2)**
**Cognitive Problems**						
Yes	69 (57.0)	15 (46.9)	**84 (54.9)**	121 (61.1)	75 (56.0)	**196 (59.0)**
No	52 (43.0)	17 (53.1)	**69 (45.1)**	77 (38.9)	59 (44.0)	**136 (41.0)**
**Depressive Symptoms**						
Yes	73 (61.3)	12 (40.0)	**85 (57.0)**	127 (64.5)	67 (50.4)	**194 (58.8)**
No	46 (38.7)	18 (60.0)	**64 (43.0)**	70 (35.5)	66 (49.6)	**136 (41.2)**
**Anxiety Symptoms**						
Yes	50 (41.7)	4 (13.3)	**54 (36.0)**	72 (36.2)	39 (29.5)	**111 (33.5)**
No	70 (58.3)	26 (86.7)	**96 (64.0)**	127 (63.8)	93 (70.5)	**220 (66.5)**
**Impulsivity Symptoms**						
Yes	82 (72.6)	14 (43.8)	**96 (66.2)**	143 (75.3)	78 (60.5)	**221 (69.3)**
No	31 (27.4)	18 (56.3)	**49 (33.8)**	47 (24.7)	51 (39.5)	**98 (30.7)**
**Physical Problems**						
Yes	115 (95.0)	24 (75.0)	**139 (90.8)**	162 (81.0)	107 (79.9)	**269 (80.5)**
No	6 (5.0)	8 (25.0)	**14 (9.2)**	38 (19.0)	27 (20.1)	**65 (19.5)**

Overall columns for primary analysis (AAS User, AAS Nonuser) are in bold.

**Table 3:** Substance use risk-taking behaviors by AAS use status.

	Current AAS User (n = 121)	Former AAS User (n = 33)	AAS User (n = 154)	AAS Nonuser, Considered Using (n = 200)	AAS Nonuser, Never Considered Using (n = 138)	AAS Nonuser (n = 338)
**N (%)**						
**Shared Needles**						
Yes	3 (2.5)	0 (0)	**3 (1.9)**	1 (0.5)	1 (0.7)	**2 (0.6)**
No	118 (97.5)	33 (100.0)	**151 (98.1)**	199 (99.5)	137 (99.3)	**336 (99.4)**
**Reused Needles**						
Yes	9 (7.4)	1 (3.0)	**10 (6.5)**	1 (0.05)	1 (0.07)	**2 (0.6)**
No	112 (92.6)	32 (97.0)	**144 (93.5)**	197 (99.5)	135 (99.3)	**332 (99.4)**
**Used Hallucinogens**						
Yes	48 (40.3)	9 (27.3)	**57 (37.5)**	55 (27.5)	17 (12.4)	**72 (21.4)**
No	71 (59.7)	24 (72.3)	**95 (62.5)**	145 (72.5)	120 (87.6)	**265 (78.6)**
**Used Stimulants**						
Yes	68 (56.2)	8 (25.0)	**76 (49.7)**	68 (34.0)	15 (10.9)	**83 (24.6)**
No	53 (43.8)	24 (75.0)	**77 (50.3)**	132 (66.0)	123 (89.1)	**255 (75.4)**
**Used Depressants**						
Yes	38 (31.4)	5 (15.2)	**43 (27.9)**	29 (14.6)	5 (3.6)	**34 (10.1)**
No	83 (68.6)	28 (84.8)	**111 (72.1)**	170 (85.4)	132 (96.4)	**302 (89.9)**
**Used Opiates**						
Yes	34 (28.1)	7 (21.2)	**41 (26.6)**	33 (16.6)	9 (6.6)	**42 (12.5)**
No	87 (71.9)	26 (78.8)	**113 (73.4)**	165 (82.9)	128 (93.4)	**293 (87.5)**
**History of Polysubstance Use (3 + of hallucinogens, stimulants, depressants, and opiates)**						
Yes	35 (29.4)	4 (12.5)	**39 (25.8)**	28 (14.2)	6 (4.4)	**34 (10.2)**
No	84 (70.6)	28 (87.5)	**112 (74.2)**	169 (85.8)	129 (95.6)	**298 (89.8)**

Overall columns for primary analysis (AAS User, AAS Nonuser) are in bold.

**Table 4:** Associations of AAS Use, APED Variety, and Number of Cycles with Traits of Interest.

Variable (reference group “No”)	AAS User Compared to AAS Nonuser	Current AAS User Compared to Former AAS User	Former AAS User Compared to AAS Nonuser, Considered Using	AAS Nonuser, Considered Using Compared to AAS Nonuser, Never Considered Using	Number of Different APED Types Used (per 1 unit increase)	Number of AAS Cycles (per 1 unit increase)
**OR (95% CI)**						
**Psychopathic Traits**						
Unadjusted	1.45 (0.96–2.17)	**2.47 (1.03–6.62)**	0.54 (0.21–1.27)	**1.93 (1.17–3.26)**	1.04 (0.96–1.14)	0.96 (0.87–1.06)
Adjusted	**2.50 (1.52–4.15)**	1.86 (0.72–5.24)	1.15 (0.41–2.95)	**2.19 (1.27–3.87)**	**1.19 (1.07–1.33)**	1.11 (0.98–1.25)
**Substance Use Risk-Taking Behavior**						
Unadjusted	**3.02 (2.04–4.51)**	1.75 (0.80–3.83)	1.31 (0.62–2.75)	**3.08 (1.88–5.16)**	**1.23 (1.13–1.35)**	**1.27 (1.14–1.46)**
Adjusted	**3.10 (1.97–4.93)**	1.90 (0.83–4.37)	1.22 (0.55–2.77)	**3.51 (2.06–6.14)**	**1.24 (1.12–1.38)**	**1.26 (1.10–1.46)**
**Sexual risk-taking behavior**						
Unadjusted	**3.07 (1.86–5.31)**	0.91 (0.25–2.70)	1.99 (0.73–6.96)	**3.11 (1.94–5.03)**	**1.31 (1.15–1.52)**	**1.40 (1.16–1.78)**
Adjusted	**1.79 (1.01–3.26)**	1.31 (0.34–4.25)	0.84 (0.28–3.14)	**3.38 (2.00–5.78)**	**1.18 (1.02–1.38)**	1.17 (0.95–1.51)
**Anger problems**						
Unadjusted	1.54 (0.97–2.50)	1.59 (0.60–3.92)	0.69 (0.30–1.75)	**2.67 (1.63–4.43)**	1.06 (0.96–1.18)	0.97 (0.89–1.07)
Adjusted	**1.71 (1.02–2.95)**	1.43 (0.53–3.66)	0.80 (0.32–2.12)	**3.16 (1.86–5.42)**	1.09 (0.97–1.23)	0.98 (0.87–1.11)
**Emotional stability problems**						
Unadjusted	1.33 (0.89–1.99)	2.17 (0.97–4.83)	0.60 (0.28–1.28)	**1.63 (1.04–2.56)**	1.09 (1.00–1.20)	1.02 (0.93–1.12)
Adjusted	1.54 (0.97–2.46)	1.73 (0.75–3.97)	0.79 (0.35–1.80)	**1.87 (1.16–3.01)**	**1.15 (1.04–1.27)**	1.05 (0.95–1.18)
**Cognitive problems**						
Unadjusted	0.85 (0.57–1.24)	1.44 (0.66–3.15)	0.56 (0.26–1.19)	1.24 (0.79–1.93)	0.95 (0.87–1.02)	0.93 (0.85–1.01)
Adjusted	1.01 (0.65–1.57)	1.25 (0.55–2.86)	0.72 (0.32–1.61)	1.53 (0.95–2.46)	0.98 (0.89–1.08)	0.99 (0.88–1.10)
**Depressive symptoms**						
Unadjusted	0.93 (0.63–1.38)	**2.38 (1.06–5.51)**	**0.37 (0.16–0.80)**	**1.79 (1.14–2.80)**	0.99 (0.91–1.07)	0.96 (0.88–1.05)
Adjusted	1.02 (0.65–1.61)	2.02 (0.86–4.86)	0.43 (0.18–1.01)	**2.12 (1.32–3.44)**	1.01 (0.92–1.11)	1.00 (0.90–1.11)
**Anxiety symptoms**						
Unadjusted	1.11 (0.74–1.67)	**4.64 (1.68–16.47)**	**0.27 (0.08–0.73)**	1.35 (0.85–2.18)	1.06 (0.98–1.15)	1.07 (0.99–1.18)
Adjusted	1.14 (0.71–1.80)	2.36 (0.81–8.66)	**0.30 (0.08–0.84)**	1.42 (0.87–2.34)	1.08 (0.98–1.19)	1.11 (1.00–1.25)
**Impulsivity symptoms**						
Unadjusted	0.87 (0.57–1.33)	**3.40 (1.52–7.78)**	**0.26 (0.12–0.55)**	**1.99 (1.23–3.23)**	0.99 (0.90–1.08)	1.02 (0.92–1.14)
Adjusted	1.01 (0.63–1.63)	**2.92 (1.27–6.84)**	**0.33 (0.14–0.74)**	**2.17 (1.31–3.61)**	1.01 (0.91–1.12)	1.07 (0.95–1.23)
**Physical problems**						
Unadjusted	**2.40 (1.34–4.59)**	**6.39 (2.04–21.06)**	0.70 (0.30–1.78)	1.08 (0.62–1.86)	**1.34 (1.14–1.64)**	**1.85 (1.31–2.97)**
Adjusted	**2.23 (1.16–4.51)**	**5.86 (1.83–19.74)**	0.63 (0.25–1.72)	1.33 (0.74–2.39)	**1.33 (1.12–1.66)**	**1.85 (1.29–3.01)**

Results with CI excluding OR = 1 (no difference) are bolded.

In secondary analyses with four levels of AAS use, AAS nonusers who considered using had higher odds of psychopathic traits (OR = 2.19, 95% CI 1.27–3.87), substance use risk-taking (OR = 3.51, 95% CI 2.06–6.14), sexual risk-taking (OR = 3.38, 95% CI 2.00–5.78), anger problems (OR = 3.16, 95% CI 1.86–5.42), emotional stability problems (OR = 1.87, 95% CI 1.16–3.01), depressive symptoms (OR = 2.12, 95% CI 1.32–3.44), and impulsivity symptoms (OR = 2.17, 95% CI 1.31–3.61) compared to AAS nonusers who never considered using; former AAS users had lower odds of both anxiety symptoms (OR = 0.30, 95% CI 0.08–0.84) and impulsivity symptoms (OR = 0.33, 95% CI 0.14–0.74) compared to AAS nonusers who considered using; and current AAS users had higher odds of both impulsivity symptoms (OR = 2.92, 95% CI 1.27–6.84) and physical problems (OR = 5.86, 95% CI 1.83–19.74) compared to former AAS users.

Lastly, we assessed possible relationships between (i) the number of different APED types used and (ii) the number of AAS cycles with the same traits of interest as before. Each additional type of APED used was associated with a 19% increase in the odds of psychopathic traits (OR = 1.19, 95% CI 1.07–1.33), a 24% increase in the odds of substance use risk-taking (OR = 1.24, 95% CI 1.12–1.38), an 18% increase in the odds of sexual risk-taking (OR = 1.18, 95% CI 1.02–1.38), a 15% increase in the odds of emotional stability problems (OR = 1.15, 95% CI 1.04–1.27), and a 33% increase in the odds of physical problems (OR = 1.33, 95% CI 1.12–1.66). For every one-unit increase in the number of AAS cycles, there was a 26% increase in the odds of substance use risk-taking (OR = 1.26, 95% CI 1.10–1.46) and an 85% increase in the odds of physical problems (OR = 1.85, 95% CI 1.29–3.01).

## Discussion

In our online survey of adult biologically male bodybuilders, we found AAS use was associated with higher odds of psychopathic traits, both for AAS users compared to nonusers as well as for increased APED variety. Importantly, this association was also present among participants with no history of polysubstance use. It is not certain whether AAS use predicts psychopathic traits or if the existence of psychopathic traits may actually be a risk factor for AAS use. We note that AAS nonusers who considered AAS use had over twice the odds of psychopathic traits compared to AAS nonusers who never considered AAS use. A recent study of 285 competitive athletes reported that Machiavellianism and psychopathy explained 29% of the variance in positive attitude toward AAS^72^. This is supported generally by the well-established association between psychopathic traits and risk-taking behaviors such as substance abuse^48^. In that case, a large proportion of bodybuilders willing to make the jump to using AAS may already have pre-existing psychopathic traits. Psychopathy is related to both antisocial personality disorder and conduct disorder, each of which is associated with AAS use^9,60^. Conduct disorder in particular is a major risk factor for AAS use^9^ that cannot be entirely explained by use of other drugs^59^. The relationship may be dynamic; bodybuilders with psychopathic tendencies may be more willing to begin AAS in the first place. Subsequently, these traits might be amplified either chemically by AAS use or psychologically by the environment; prior work has shown the difference between psychopaths and non-psychopaths in emotional-regulatory activity in the aPFC is modified by endogenous testosterone level^73^. With this in mind, longitudinal research is needed to further explore the causal nature of this relationship.

Our study is one of many to link AAS use substance use risk-taking behaviors^74,75^ and sexual risk-taking behaviors^59,76^. It is difficult to ascertain the specific relationship between AAS use and risk-taking. Unlike physical, psychological, cognitive, and anger problems, which have all had experimental and translational research done to strengthen causal interpretations of such links^16,77^, there has not been experimental work to test whether risk-taking behaviors are caused by AAS use. In fact, it is important to consider that AAS use is itself a risk behavior, and another form of substance use, so AAS users may already engage in many other risk-taking behaviors prior to their first use. This may be especially true in light of our findings that AAS nonusers who considered AAS use had over three-times the odds of both substance use and sexual risk-taking behaviors compared to AAS nonusers who never considered AAS use, as well as our results regarding APED variety and AAS cycle experience. AAS users willing to try more types of APEDs or willing to undergo more AAS cycles may be more likely to also engage in risk-taking behaviors. Perhaps the relationship between AAS and risk-taking behaviors is bidirectional and interactive, where athletes that engage in these risk behaviors such as illicit drug use experiment with AAS, which may lower their inhibitions to take further risks.

Our finding that AAS users have higher odds of experiencing anger problems is in line with prior research^16,19,20^. Notably, anger has been previously reported as both a potential risk factor^78^ as well as a potential outcome^27^. We did not observe associations between AAS use and emotional stability problems, cognitive problems, depressive symptoms, anxiety symptoms, or impulsivity symptoms. Prior research has identified various psychological and cognitive traits among AAS users such as depression, impulsivity, and mania^18–20^, but they are generally idiosyncratic in nature^8,79–81^. We do note that AAS nonusers who considered AAS use had higher odds of emotional stability problems, depressive symptoms, and impulsivity symptoms compared to AAS nonusers who never considered AAS use, former AAS users had lower odds of anxiety symptoms and impulsivity symptoms compared to AAS nonusers who considered AAS use, and current AAS users had higher odds of impulsivity symptoms compared to former AAS users. These findings comparing AAS nonusers who considered vs. never considered AAS use are consistent with prior research about factors relating to the decision to use AAS, including research on the “Big Five” personality traits^58^. Additionally, we observed increased odds of emotional stability problems with increased APED variety. Lastly, our hypothesis about physical problems was supported for AAS users compared to nonusers as well as the dose dependent response in relation to increased APED variety and increased AAS cycle experience. These findings are consistent with prior studies^3,8,10,32^. 

There are several limitations. Although we successfully elicited responses from real-world users of AAS, there remain questions about how representative our sample is. AAS users in our sample were relatively new users (median of 2 prior cycles). Our findings may have been different with a group of more experienced users. It is also possible that our online survey was more likely to attract individuals with psychopathic traits or that AAS users with psychopathic traits are more willing to take an online survey than other users. We note that > 50% of AAS users and nonusers were considered to have substance use risk-taking, sexual risk-taking, anger problems, emotional stability problems, cognitive problems, depressive symptoms, impulsivity symptoms, and physical problems. Lastly, this cross-sectional study is entirely correlational and any attempts to speculate about causality should be made with extreme caution. Further prospective or experimental studies are needed. In light of the findings on Machiavellianism and psychopathy in relation to willingness to use AAS^72^, it would be interesting to also examine the link to narcissism and self-esteem/insecurity^82^. We wonder whether self-esteem or narcissistic traits could play an additional role in the motivation to begin AAS use, given the known downsides.

This study is among the first to directly assess psychopathy within AAS users. Our results on risk-taking, anger problems, and physical problems are consistent with prior AAS research as well as with existing frameworks of AAS use as a risk behavior. Increased psychopathic traits in AAS users may serve as the underlying mechanism to predict increased anger problems (see^60^ regarding antisocial personality disorder as a mechanism between AAS and aggression). Although the present study highlights the relationship between AAS use and psychopathic traits, future research should emphasize possible causal explanations and try to elucidate the directionality of this relationship. Additionally, the mechanisms between AAS use and risk and violent behaviors should be further explored.

## Supplementary Information

Supplementary Information 1.

Supplementary Information 2.

## Data Availability

All data generated or analyzed during this study are included in this published article’s supplementary information files. R code used in data analysis can be made available upon reasonable request to the corresponding author.
